# Small Extracellular Vesicles of M1-BV2 Microglia Induce Neuronal PC12 Cells Apoptosis via the Competing Endogenous Mechanism of CircRNAs

**DOI:** 10.3390/genes13091603

**Published:** 2022-09-08

**Authors:** Sheng Gao, Luyue Bai, Shu Jia, Chunyang Meng

**Affiliations:** 1 Department of Medicine, Qingdao University, Qingdao 266071, China; 2 Department of Medicine, Jining Medical University, Jining 272067, China

**Keywords:** apoptosis, circRNAs, microglia, neuronal cells, extracellular vesicles

## Abstract

Polarized microglia play a vital role in neurodegenerative diseases. However, the effects of polarized microglia-derived small extracellular vesicles (SEVs) on neuronal cells and the regulatory mechanisms of circular RNAs (circRNAs) in SEVs remain incompletely defined. In the present study, we carried out high-throughput sequencing and differential expression analysis of circRNAs in the SEVs of M0-phenotype BV2 microglia (M0-BV2) and polarized M1-phenotype BV2 microglia (M1-BV2). Hub circRNAs in the SEVs and their functions were screened using multiple bioinformatics methods. We further validated the effects of SEVs on neuronal PC12 cells by co-culturing M0-BV2 SEVs and M1-BV2 SEVs with neuronal PC12 cells. Among the differentially expressed circRNAs, the target mRNAs of six hub circRNAs (circ_0000705, circ_0001313, circ_0000229, circ_0001123, circ_0000621, and circ_0000735) were enriched in apoptosis-related biological processes. Furthermore, western blot and flow cytometry analysis demonstrated that M0-BV2 SEVs had no distinct effect on apoptosis of neuronal PC12 cells, while M1-BV2 SEVs remarkably increased the apoptosis of neuronal PC12 cells. We then constructed the competing endogenous RNA (ceRNA) networks of the six hub circRNAs. Taken together, the results suggest that polarized M1-BV2 microglia can induce apoptosis of neuronal PC12 cells through secreted SEVs, and this regulatory effect may be achieved by the circRNAs circ_0000705, circ_0001313, circ_0000229, circ_0001123, circ_0000621, and circ_0000735 through ceRNAs regulatory networks. These findings provide new potential targets for the treatment of neurodegenerative diseases.

## 1. Introduction

Neurodegenerative diseases are highly disabling and ultimately fatal conditions affecting millions of individuals worldwide; however, no curative therapies are currently available to arrest or reverse their progression [1]. It is now recognized that microglia are involved in the occurrence and progression of neurodegenerative diseases [2,3]. Microglia account for approximately 12% of the total cellular population in the mammalian brain [4], and have two main polarized forms: M1 and M2 [5]. M1 phenotype microglia exert a pro-inflammatory effect primarily by expressing IL-6, IL-1β, and TNF-α [6,7], whereas M2 phenotype microglia exert an anti-inflammatory response by secreting IL-4, IL-10, and TGF-β [8]. As a result, there is substantial variation in the impact of microglia on the central nervous system (CNS). Injury stimulus can induce resting M0 phenotype microglia polarized into M1 phenotype microglia [9], eventually leading to irreversible neuron loss [10]. The effects of M1 microglia on the CNS have been widely reported in neuro-immuno-inflammatory studies [11,12]; however, research on the effects of M1 microglia-derived extracellular vesicles (EVs) on neuronal cells is rare. EVs first form as intraluminal vesicles in multivesicular compartments and are either secreted upon fusion of these compartments with the plasma membrane or released directly from the plasma membrane [13]. Small extracellular vesicles (SEVs), a type of EV measuring 30–100 nm in diameter, function as important cell–cell messengers [14,15].

Several recent studies have shown that non-coding RNAs in SEVs can affect intercellular communication, signal transduction, and metabolic regulation [16,17,18]. CircRNAs, a kind of highly conserved, endogenous, non-coding RNA, are important regulators of intercellular information. They are resistant to cleavage by exonuclease due to their unique property of lacking a 5′ cap and 3′ tail, which makes them more stable than linear RNAs [19,20]. In addition, circRNAs can lower the level of miRNAs that act as “miRNA sponges” that release downstream target mRNAs by lowering the level of miRNAs [21,22,23]. Compared to other ncRNAs, circRNAs have unique characteristics that include stability and high enrichment in SEVs [24,25]. However, the effects of microglia-derived SEVs on neuronal cells and the potential regulatory mechanism of circRNAs in SEVs remain undefined. The present study aimed to explore the effect of polarized M1 phenotype microglial SEVs on neuronal cells and to construct the competing endogenous RNAs (ceRNAs) regulatory networks of hub circRNAs in SEVs, thereby identifying potential new targets for the treatment of neurodegenerative diseases.

## 2. Materials and Methods

### 2.1. Culture of BV2 Microglial Cells

BV2 microglial cells were purchased from Procell Life Science & Technology Co., Ltd. (Wuhan, China) and cultured in Dulbecco’s Modified Eagle Medium (DMEM) supplemented with 10% fetal bovine serum (FBS) and 100 U/mL penicillin/streptomycin at 37 °C under 5% CO_2_. The cells were passaged with 0.25% trypsin-EDTA (Gibco, Grand Island, NY, USA) when they attained 80% confluence.

### 2.2. Induction and Identification of BV2 Microglial Cells

BV2 microglial cells (M0 phenotype) were divided into two groups, and inoculated in six-well plates. The resting M0-BV2 microglial cells in the control group were untreated (M0-BV2 group), while those in the test group were treated with 1 μg/mL lipopolysaccharide (LPS; *Escherichia coli* LPS 055: B5, Sigma Aldrich, Saint-Louis, MO, USA) for 12 h to induce polarization into the M1 phenotype (M1-BV2 group). Two groups of BV2 microglial cells were incubated with CD40 (BioLegend, San Diego, CA, USA) and CD86 (BioLegend, San Diego, CA, USA), which are M1 phenotype biomarkers, at room temperature for 15 min and then assessed by flow cytometry. These M1 phenotype biomarkers demonstrated successful induction of M1-BV2 microglial cells.

### 2.3. Extraction and Detection of SEVs from BV2 Microglial Cells

BV2 microglial cells were sub-cultured until the total number of cells was approximately 1.8 × 10^8^. Half of these cells then were induced to the M1 phenotype with 1 μg/mL LPS (*EC* LPS 055: B5, Sigma-Aldrich, Saint-Louis, MO, USA) for 12 h. The cells were washed with PBS three times in the culture flask and then cultured in DMEM supplemented with 10% FBS (without EVs) for 48 h. The supernatant of M0-BV2 and M1-BV2 microglial cells (approximately 135 mL per group) was collected in centrifuge tubes and centrifuged at 300× *g* at 4 °C for 10 min. The supernatant obtained was then centrifuged at 2000× *g* at 4 °C for 10 min. To remove any remaining large EVs, the collected supernatant was further transferred into high-speed centrifuge tubes and centrifuged at 10,000× *g* at 4 °C for 30 min. The concentrated supernatant was then aseptically filtered with a 0.22 μm needle sterilizing filter (Millipore Sigma, Darmstadt, Hessen, Germany). We added the filtered supernatant to high-speed centrifuge tubes, which were then centrifuged at 100,000× *g* at 4 °C for 70 min to collect the SEVs.

Nanoparticle tracking analysis (NTA) was used to evaluate the concentration and diameter of M0-BV2 SEVs and M1-BV2 SEVs. The morphology of SEVs was analyzed using a transmission electron microscope (TEM). The protein markers Tsg101 (1:1000; ABclonal) and Alix (1:1000; CST) of SEVs were identified by western blot.

### 2.4. High-Throughput Sequencing of circRNAs in M0-BV2 SEVs and M1-BV2 SEVs

The total RNA of M0-BV2 and M1-BV2 SEVs was extracted using Invitrogen TRIzol LS (Invitrogen Corp, Carlsbad, CA, USA). The NEBNext^®^ rRNA Depletion Kit (NEB, E7400S) was used to digest ribosomal RNA (rRNA). Linear RNA was then digested using RNase R (Epicentre, RNR07250) following the manufacturer’s instructions. The library was constructed using the KAPA Stranded RNA-Seq Library Prep Kit (Illumina). Quality analysis of the library was performed using Agilent 2100. Next, the generated fragment was sequenced using an Illumina HiSeq 4000 sequencer. The circRNAs were aligned to the reference genome using STAR software and Backsplice junction reads were tested by LNCexplorer2.

### 2.5. Screening of Hub circRNAs

Differential expression of circRNAs in M0-BV2 and M1-BV2 SEVs was analyzed using DESeq (v1.18.0; https://bioconductor.org/packages/release/bioc/html/DESeq.html, accessed on 29 June 2021), and the differentially expressed circRNAs (DEcircRNAs) in the exosomes were screened with a *p* < 0.01 and |log2FC| > 0.585 (fold change ≥ +1.5 or fold change ≤ −1.5) as the threshold. To further explore the hub circRNAs in SEVs and the ceRNAs involved in regulatory networks (circRNA:miRNA:mRNA), DEcircRNAs with miRNA binding sites (MREs) and bound miRNAs were screened by starBase (http://starbase.sysu.edu.cn/, accessed on 29 June 2021). Furthermore, the target mRNAs bound by the miRNAs were obtained using miRNet (https://www.mirnet.ca/miRNet/home.xhtml, accessed on 29 June 2021), a comprehensive miRNA-target prediction database.

### 2.6. Enrichment Analysis and ceRNA Regulatory Networks

To define the main molecular functions and pathways regulated by the hub circRNAs of SEVs, we performed Gene Ontology (GO) and Kyoto Encyclopedia of Genes and Genomes (KEGG) pathway enrichment analysis of the target mRNAs in the ceRNAs regulatory networks. GO enrichment analysis was performed using the molecular function (MF), cell composition (CC), and biological process (BP) functional terms. Significantly enriched GO entries and KEGG pathways were obtained using *p* < 0.05 as the threshold. The enrichment results revealed significant enrichment of apoptosis-related biological processes. We then constructed ceRNAs regulatory networks of circRNA:miRNA:mRNA in apoptosis-related molecular functions using Cytoscape (Version: 3.80).

### 2.7. Labeling of BV2 Microglia SEVs

The M0-BV2 and M1-BV2 SEVs were stained with 1 µM 1,1′-dioctadecyl-3,3,3′,3′-tetramethylindocarbocyanine perchlorate (DiI; ATT Bioques, Sunnyvale, CA, USA) for 30 min. They were then washed in PBS at 100,000× *g* for 2 h to remove excess dye according to previous studies [26,27]. The DiI-labeled SEVs were finally obtained after resuspension in PBS.

### 2.8. Culture and Treatment of Neuronal PC12 Cells

The PC12 cell line was obtained from the Cell Resource Center of the Institute of Basic Medical Sciences, Chinese Academy of Medical Sciences. The cells were cultured in RPMI-1640 supplemented with 10% donor horse serum, 5% FBS and 100 U/mL penicillin/streptomycin at 37 °C under 5% CO_2_, and passaged with 0.25% trypsin-EDTA. Differentiation of the PC12 cell line into neuronal PC12 cells (neuron-like cells with neuronal characteristics) was induced using 50 ng/mL nerve growth factor (Sigma-Aldrich, Saint Louis, MO, USA) for 14 days, according to prior studies [28]. Neuronal PC12 cells were inoculated into a six-well plate and divided into a control group, M0-BV2-SEVs group, and M1-BV2-SEVs group. Neuronal PC12 cells in the M0-BV2-SEVs group and M1-BV2-SEVs group were added to M0-BV2 SEVs (10^10^ particles) and M1-BV2 SEVs (10^10^ particles), respectively, while those in the control group were treated with the same volume of PBS. The original medium was replaced with a fresh medium (2.5 mL/well) to remove any SEVs secreted by neuronal PC12 cells before the addition of M0-BV2 SEVs, M1-BV2 SEVs, or PBS. Subsequently, the two kinds of BV2 SEVs were co-incubated with neuronal PC12 cells for 24 h.

### 2.9. Immunofluorescence Analysis

After 24 h, the neuronal PC12 cells in the three treatment groups were stained using a 1 µM SiR700-Tubulin Kit (Spirochrome, Stein am Rhein, Switzerland) and 1 µM Hoechst (Thermo Fisher Scientific, Waltham, MA, USA) at 37 °C for 30 min in a 5% CO_2_ incubator, following the manufacturer’s instructions. The entry of DiI-labeled (ATT Bioques, Sunnyvale, CA, USA) SEVs into cells and morphological changes to neuronal PC12 cells in the control, M0-BV2-SEVs, and M1-BV2-SEVs groups were analyzed using a confocal microscope (Zeiss LSM800; Zeiss AG; magnification, ×630). Internalization of SEVs was quantified using ImageJ software with the relative fluorescence intensity of SEVs per cell (SEVs/cell), which were calculated with integrated density/cells. Quantitative analysis of the number and length of neurites was performed using the ImageJ software with the NeuronJ and Sholl analysis plugin, which were developed for the quantitative analysis of neurites.

### 2.10. Protein Extraction, Determination, and Western Blotting

The neuronal PC12 cells were first detached with 0.25% trypsin-EDTA and collected by centrifugation at 300× *g* for 5 min. The cells were then washed with PBS three times and collected by centrifugation at 300× *g* for 5 min. Next, 150 μL of the protein extract RIPA (Beyotime, Shanghai, China) was added to the cells in each group and fully lysed on ice for 30 min. The supernatant was collected by centrifugation at 12,000× *g* for 15 min at 4 °C. The extracted protein was determined using the BCA kit (Beyotime, Shanghai, China) following the manufacturer’s protocol. Subsequently, a 25 μg protein sample from the control, M0-BV2-SEVs, or M1-BV2-SEVs group was separated in 12% SDS-PAGE (Bio-Rad Laboratories, Hercules, CA, USA) and transferred to a polyvinylidene fluoride (PVDF) membrane at 300 mA. The blots were blocked with 3% bovine serum albumin (Beyotime, Shanghai, China) for 2 h and then incubated with Bak (1:1000; CST), Bcl-XL (1:1000; CST), and Cleaved Caspase-3 (1:1000; CST) primary antibodies at 4 °C overnight. Afterwards, the samples were washed three times with Tris-Buffered Saline Tween-20 (TBST) and incubated with HRP-conjugated secondary antibody (1:4000; goat anti-rabbit IgG, ABclonal) at room temperature for 1 h. The protein bands were exposed to ECL reagent (Beyotime, Shanghai, China) and visualized using a chemiluminescent detector (Tanon Science and Technology Co., Ltd., Shanghai, China). Expression was quantified using ImageJ software.

### 2.11. Flow Cytometry Analysis

The neuronal PC12 cells in the three groups were washed twice with pre-cooled PBS and processed using the Annexin V-APC/7AAD kit (BioLegend, San Diego, CA, USA) following the manufacturer’s instructions. Briefly, the cells were digested with EDTA-free trypsin (Solarbio, Beijing, China). After incubation with stain and binding buffer at room temperature for 15 min in darkness, the mixed cell suspension was transferred to flow tubes. The apoptosis rate was then determined using a flow cytometer (CytoFlex; Beckman Coulter, Inc., Brea, CA, USA).

### 2.12. MTT Assay

The neuronal PC12 cells were cultivated in 96-well plates at a density of 1 × 10^4^ cells/well. Cells treated with PBS, M0-BV2-SEVs, or M1-BV2-SEVs for 24 h were incubated with 10 µL MTT (Beyotime, Shanghai, China) solution (5 mg/mL) for 4 h at 37 °C. A total of 100 µL formazan solution was added to each well and incubated for 3 h at 37 °C, according to the manufacturer’s instructions. Absorbance was measured at 570 nm using an automatic microplate reader. Cell viability was shown as a percentage of the control.

### 2.13. Statistical Analysis

Data are presented as the mean ± standard deviation (SD) for three independent experiments. Comparisons were made using an unpaired Student’s *t*-test or one-way analysis of variance (ANOVA). Values of *p* < 0.05 were considered statistically significant. GraphPad Prism 8 (GraphPad Software, Inc., San Diego, CA, USA) was used to perform the statistical analysis. The upregulated and downregulated circRNAs were screened with a fold change ≥ +1.5 or fold change ≤ −1.5 as the threshold and calculated with the R package “edger” in R software (version 4.0.0).

## 3. Results

### 3.1. Identification of M0-BV2 and M1-BV2 Microglial Cells

**Figure 1:**
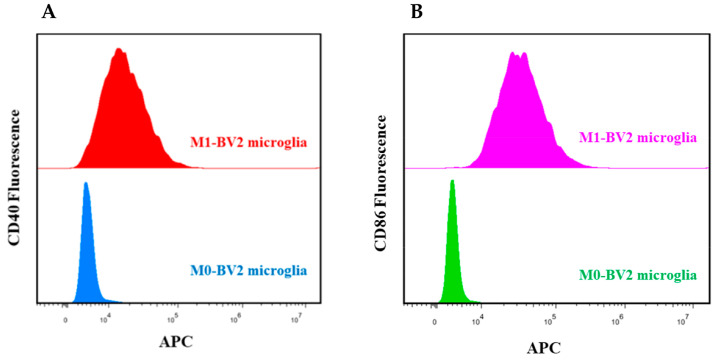
Identification of M0-BV2 and M1-BV2 microglial cells. (**A**,**B**) Flow cytometry analysis showed that CD40 and CD86 were suppressed in the M0-BV2 microglia group and overexpressed in the M1-BV2 microglia group.

Flow cytometry analysis revealed that LPS-activated M1-BV2 microglia significantly expressed CD40 and CD86, which are M1 phenotype biomarkers, whereas M0-BV2 microglia did not (Figure 1A,B). These results suggest successful polarization of M1 phenotype microglial cells.

### 3.2. Identification of BV2 Microglia-Derived SEVs

**Figure 2:**
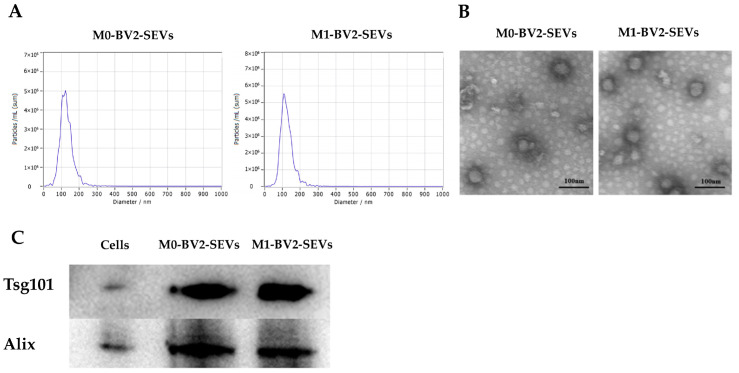
Identification of M0-BV2 and M1-BV2 SEVs. (**A**) The diameter of the extracted M0-BV2 and M1-BV2 SEVs was about 100 nm. (**B**) The structure of extracted M0-BV2 and M1-BV2 SEVs under a transmission electron microscope. Scale bar: 100 nm. (**C**) Western blots showed that protein markers (Tsg101 and Alix) of SEVs were expressed.

M0-BV2 and M1-BV2 SEVs were extracted by differential centrifugation and then analyzed. The concentration and diameter of the extract were measured using NTA, and it was found that the extract met the criteria of SEV diameter (Figure 2A). TEM revealed that the extract had a layer membrane structure (Figure 2B), which is a typical morphological feature of SEVs. In addition, the western blots demonstrated that SEVs protein markers were significantly expressed in the extract (Figure 2C).

### 3.3. High-Throughput Sequencing of circRNAs

**Figure 3:**
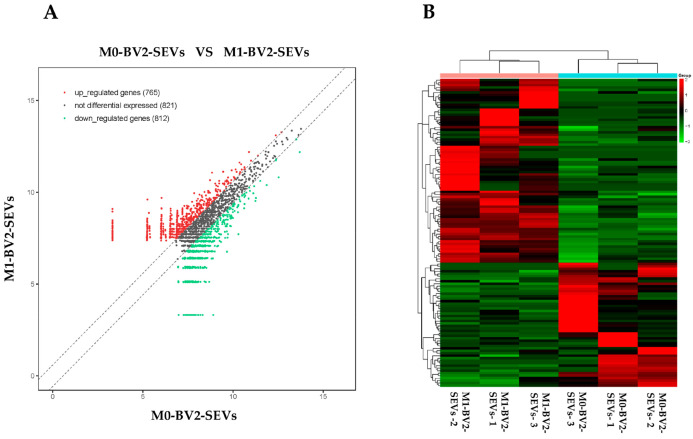
High-throughput sequencing results of circRNAs in M0-BV2 SEVs (M0-BV2-SEVs) and the M1-BV2 SEVs (M1-BV2-SEVs). (**A**) The scatterplot generated using the R package “ggplot2” showed a total of 765 upregulated (red), 821 non-difference (black), and 812 downregulated (green) circRNAs. (**B**) The heat map generated using the R package “pheatmap” showed a clustering relationship of circRNAs expression.

High-throughput sequencing was performed on the circRNAs in M0-BV2 and M1-BV2 SEVs. A total of 2398 circRNAs were obtained, including 765 upregulated (fold change ≥ +1.5), 821 non-difference, and 812 downregulated (fold change ≤ −1.5) circRNAs (Figure 3A,B).

### 3.4. Hub circRNAs and Target miRNAs

**Figure 4:**
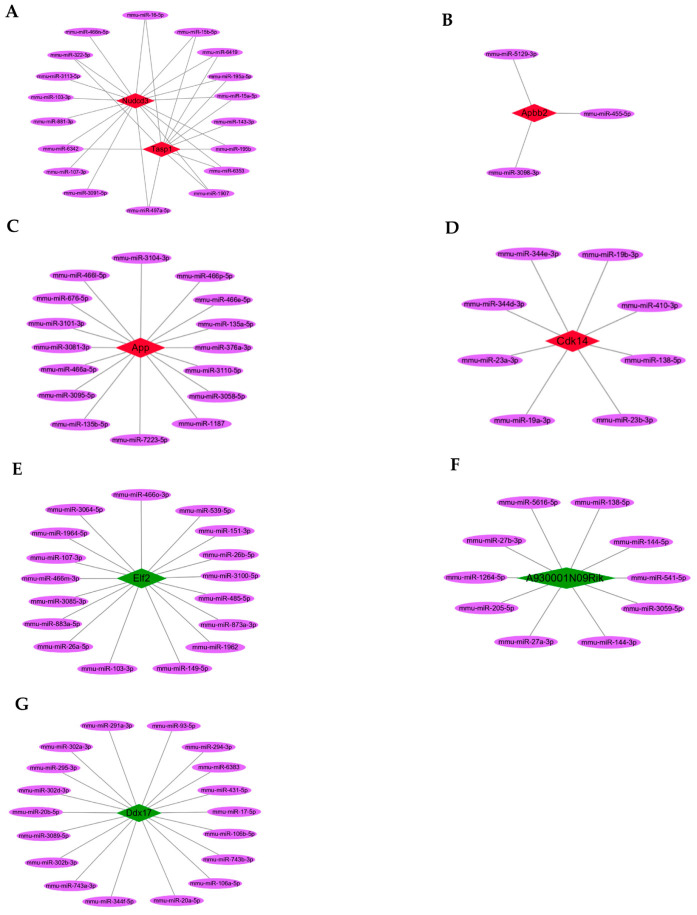
The hub circRNAs and target miRNAs. (**A**–**D**) The five upregulated hub circRNAs (red) and target miRNAs (purple). (**E**–**G**) The three downregulated hub circRNAs (green) and target miRNAs (purple).

Among the screened DEcircRNAs, five upregulated hub circRNAs (*p* < 0.01 and fold change ≥ +1.5; Table 1) could sponge 36 miRNAs (Figure 4A–D), preventing them from binding 1651 target mRNAs (Supplementary Table S1), while three downregulated hub circRNAs (*p* < 0.01 and fold change ≤ −1.5; Table 2) could sponge 41 miRNAs (Figure 4E–G), preventing them from binding 2321 target mRNAs (Supplementary Table S2).

### 3.5. GO and KEGG Pathway Enrichment Analysis

**Figure 5:**
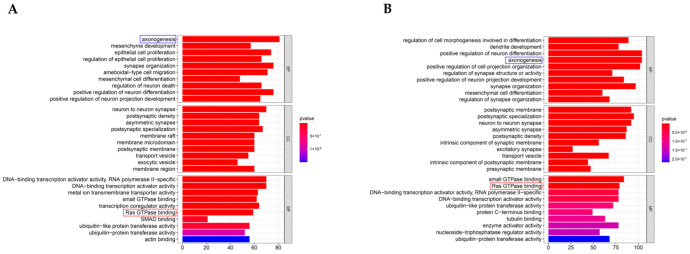
GO enrichment results. (**A**) The first 10 GO (CC, BP, and MF) enrichment results of the upregulated hub circRNAs. (**B**) The first 10 GO enrichment results of the downregulated hub circRNAs.

**Figure 6:**
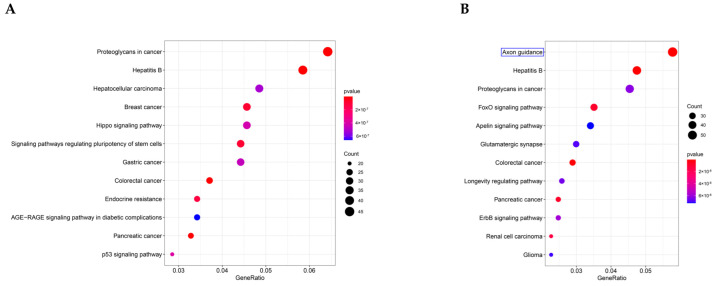
KEGG pathway enrichment results. (**A**) The first 10 enrichment pathways of the upregulated hub circRNAs. (**B**) The first 10 enrichment pathways of the downregulated hub circRNAs.

To define the main molecular functions and pathways regulated by the hub circRNAs of SEVs, we performed GO and KEGG pathway enrichment analysis. The first 10 GO entries (Figure 5A,B) and KEGG signaling pathways (Figure 6A,B) in the enrichment analysis were graphically displayed using R software (version 4.0.0). The enrichment results demonstrated that the mRNAs targeted by the three upregulated hub circRNAs (circ_0000705, circ_0001313, and circ_0000229) and downregulated hub circRNAs (circ_0001123, circ_0000621, and circ_0000735) were enriched in the entries of axon-related changes (Figure 5A,B and Figure 6B; blue mark; axonogenesis and axon guidance) and biological process of Ras GTPase binding (Figure 5A,B; red mark; Ras GTPase binding), which is a molecular function related to apoptosis [29,30].

### 3.6. ceRNAs Regulatory Networks

**Figure 7:**
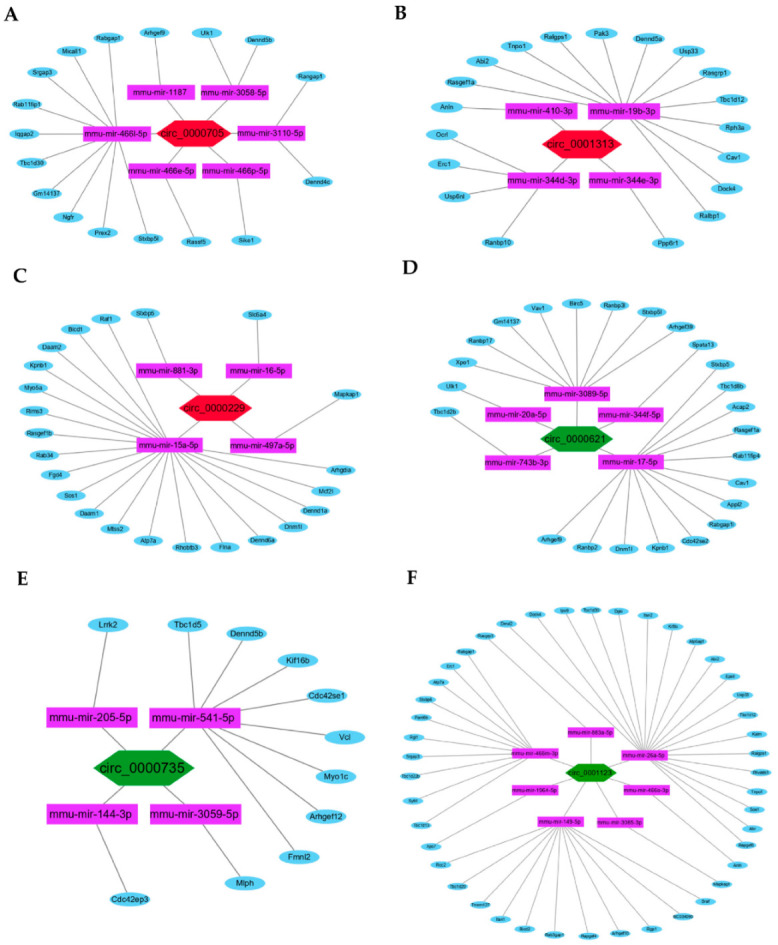
The ceRNAs regulatory networks of hub circRNAs. (**A**–**C**) The constructed ceRNAs regulatory networks of the three upregulated hub circRNAs (red): circ_0000705, circ_0001313, and circ_0000229. (**D**–**F**) The ceRNAs regulatory networks of the three downregulated hub circRNAs (green): circ_0000621, circ_0000735, and circ_0001123.

To further identify the hub circRNAs-mediated sponge regulatory mechanism, the ceRNAs regulatory networks of circRNA:miRNA:mRNA in the biological process of Ras GTPase binding were constructed and visualized using Cytoscape (Figure 7A–F).

### 3.7. Internalization of SEVs and Change to Neurites

**Figure 8:**
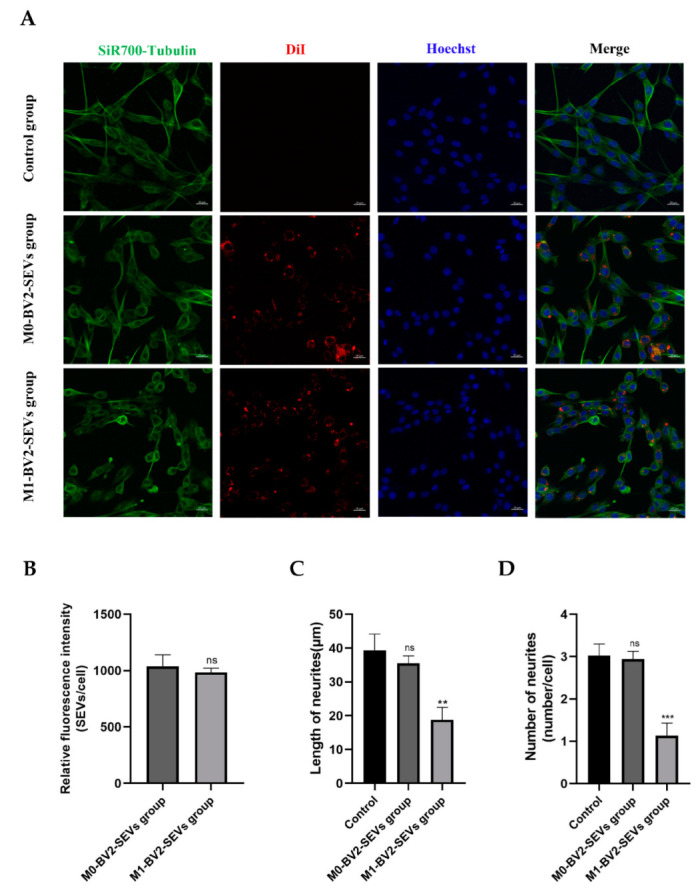
Internalization of SEVs and changes to neurites. (**A**) M0-BV2 and M1-BV2 SEVs were stained with DiI (red), and the cell cytoskeleton (green) and nucleus (blue) were stained with the SiR700-tubulin Kit and Hoechst, respectively. Scale bar: 20 μm. (**B**–**D**) Quantitative analysis of the internalization of SEVs, as well as the length and number of neurites. Data are presented as the means ± SD of three independent experiments (** *p* < 0.01, *** *p* < 0.001).

Immunofluorescence results showed the neuronal PC12 cells take up BV2 microglia-derived SEVs (Figure 8A; red). The neurites of PC12 cells in the control and M0-BV2-SEVs groups showed no obvious changes, while those in the M1-BV2-SEVs group had shortened or decreased neurites (Figure 8A; green). Quantitative analysis showed the internalization of SEVs (Figure 8B), and the length and number of neurites (Figure 8C,D).

### 3.8. M1-BV2 SEVs Induce the Apoptosis of Neuronal PC12 Cells

**Figure 9:**
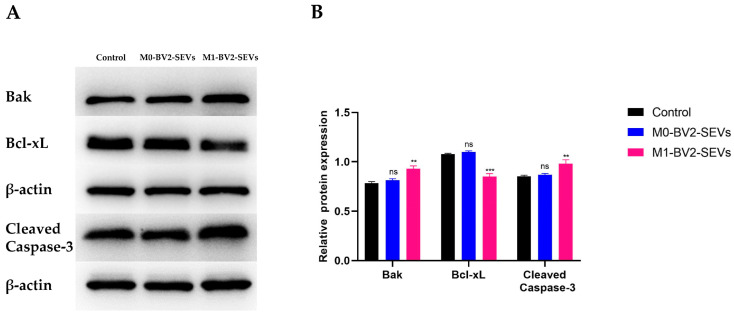
Expression of apoptosis-related proteins in neuronal PC12 cells following different treatments. (**A**) Expression of the apoptosis-related proteins Bak, Bcl-XL, and Cleaved Caspase-3 in the control, M0-BV2-SEVs, and M1-BV2-SEVs groups. (**B**) Relative expression of proteins from the western blot assay. Data are presented as the means ± SD of three independent experiments (** *p* < 0.01, *** *p* < 0.001).

**Figure 10:**
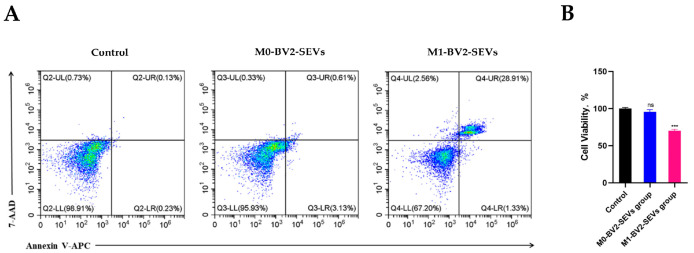
The apoptosis rate and viability assay of neuronal PC12 cells following different treatments. (**A**) Results for the apoptosis rate of neuronal PC12 cells in the control, M0-BV2-SEVs, and M1-BV2-SEVs groups. (**B**) Cell viability results of the control, M0-BV2-SEVs, and M1-BV2-SEVs groups obtained using MTT assay. Data are presented as the means ± SD of three independent experiments (*** *p* < 0.001).

Western blot analysis showed that M0-BV2 SEVs had no significant effect on the apoptosis of neuronal PC12 cells compared with the control group. In contrast, M1-BV2 SEVs significantly increased the levels of pro-apoptotic protein Bak and Cleaved Caspase-3 and suppressed the level of Bcl-xL, an anti-apoptotic protein (Figure 9A,B). Moreover, flow cytometry analysis and the MTT assay demonstrated that M0-BV2 SEVs had no significant effect on the apoptosis and proliferation of neuronal PC12 cells, whereas M1-BV2 SEVs enhanced the apoptosis rate and inhibited the proliferation of neuronal PC12 cells (Figure 10A,B).

## 4. Discussion

Neurodegenerative diseases are characterized by degeneration of distinct neuron subsets and pathological changes in specific brain areas [31]. Although the etiology of neurodegenerative diseases is multifactorial, apoptosis plays a key role in disease progression [31,32]. Apoptosis is characterized by a series of distinct biochemical alterations, such as chromatin condensation, nuclear fragmentation, dilatation of the endoplasmic reticulum, and condensation of cytoplasmic organelles [33]. The increased apoptotic rate in neurodegenerative diseases may cause consequent brain tissue damage. A previous study demonstrated that continuous normal crosstalk between neurons and microglia contributes to the homeostasis of CNS [34]. The polarization of microglia in CNS is recognized to play a major role in the occurrence and maintenance of neurodegenerative diseases [10,35].

At present, SEVs have been shown to be crucial to microglia–neuron relationships [36,37]. Microglia can communicate with neurons through secretion of SEVs. The secreted SEVs of microglia can participate in the metabolism of neurons, regulating the synaptic activity and neuronal survival [38]. Recent studies have shown that SEVs released by pro-inflammatory microglia (M1 phenotype) have been shown to contribute to the progressive neuroinflammatory response and thus drive neurodegeneration [39]. SEVs from M1 microglia can also block remyelination in neurodegenerative diseases [38]. We found that SEVs released by activated M1-BV2 microglia can shorten and decrease the number of neurites, which may further induce neurological dysfunction. Understanding the specific role of SEVs in M1 microglia is thus crucial for fully understanding the pathogenesis of neurodegenerative diseases.

Studies have implicated miR-146a-5p in neurodegenerative diseases [40]. Inflammatory microglia (M1) SEVs can transfer miR-146a-5p to neurons and downregulated synaptic targets, thus having a significant impact on synapse stability [41]. At present, many studies have shown that circRNAs, a kind of non-coding RNAs, have important effects in the progression of many complex human diseases [42,43,44]. Furthermore, circRNAs are specifically enriched in SEVs, and regulate the development of neurodegenerative diseases [45]. In recent years, various circRNAs in SEVs and their functions have been identified [46,47,48]. Thus, we constructed ceRNAs regulatory networks of circRNAs. The results showed that the hub circRNAs circ_0000705, circ_0001313, circ_0000229, circ_0001123, circ_0000621, and circ_0000735 in the SEVs of M1-phenotype microglia may mediate neuronal apoptosis through the constructed ceRNAs regulatory networks. Among these hub circRNAs, the functions of circ_0000705, circ_0001123, and circ_0000621 were not fully identified. Circ-0001313 and circ_0000735 are reported play important roles in apoptosis in human cancers, while circ_0000229 is a potential therapeutic target for glioma in the nervous system. These hub circRNAs in microglia-derived SEVs and their ceRNAs regulatory networks may provide novel targets for the treatment of neurodegenerative diseases.

Next, we further detected the expression levels of apoptosis-related proteins after co-culture of M0-BV2 or M1-BV2 SEVs with neuronal PC12 cells. We demonstrated that M0-BV2 SEVs had no significant effect on the apoptosis of neuronal PC12 cells, while M1-BV2 SEVs significantly increased the levels of pro-apoptotic proteins Bak and Cleaved Caspase-3 and decreased the level of anti-apoptotic protein Bcl-xL. The MTT assay and flow cytometry analysis also showed that M1-BV2 SEVs striking inhibited cell proliferation and increased the apoptosis rate of neuronal PC12 cells.

These findings further demonstrate that polarized M1-BV2 microglial cells can increase the apoptosis of neuronal PC12 cells through secreted SEVs, and that this regulatory effect may be related to the upregulation of circ_0000705, circ_0001313, and circ_0000229, as well as the downregulation of circ_0001123, circ_0000621, and circ_0000735 in SEVs. This study offers important new insights into the regulatory role of microglia-derived SEVs in neuronal cells. To the best of our knowledge, similar reports are rare. However, the underlying mechanism warrants further exploration using other biological methods and in vitro/vivo experiments.

## 5. Conclusions

Taken together, the results of our study demonstrated that polarized M1-BV2 microglia can induce apoptosis of neuronal PC12 cells through secreted SEVs. This regulatory effect may be achieved by circRNAs (circ_0000705, circ_0001313, circ_0000229, circ_0001123, circ_0000621, and circ_0000735) in SEVs through ceRNAs regulatory networks. These findings may serve as a basis for identifying new potential therapeutic targets for neurodegenerative diseases.

## Figures and Tables

**Table 1:** The upregulated hub circRNAs.

ID	Name	log2FC	Fold Change	*p* Value	*q* Value
circ_0001352	Apbb2	1.660178807	3.16055694	0.000921133	0.21954373
circ_0000705	App	5.633184648	49.63151705	0.000914221	0.21954373
circ_0001313	Cdk14	3.649651721	12.55031544	0.000217361	0.089744752
circ_0000229	Nudcd3	1.936416359	3.827537094	0.001007081	0.21954373
circ_0001073	Tasp1	5.160849096	35.7742371	0.008136301	0.746455744

**Table 2:** The downregulated hub circRNAs.

ID	Name	log2FC	Fold Change	*p* Value	*q* Value
circ_0001123	Elf2	−1.477538223	0.35910105	0.001406951	0.240990559
circ_0000621	Ddx17	−5.586364347	0.0208131	0.001883986	0.301186517
circ_0000735	A930001N09Rik	−1.713429367	0.304934361	0.000164008	0.089744752

## Data Availability

Not applicable.

## Supplementary Materials

The following supporting information can be downloaded at: https://www.mdpi.com/article/10.3390/genes13091603/s1, Table S1: Target mRNAs in the regulatory networks of upregulated hub circRNAs; Table S2: Target mRNAs in the regulatory networks of downregulated hub circRNAs.
